# How general practitioners understand and handle medically unexplained symptoms: a focus group study

**DOI:** 10.1186/s12875-018-0745-2

**Published:** 2018-05-02

**Authors:** Erik Børve Rasmussen, Karin Isaksson Rø

**Affiliations:** 1 Centre for the study of professions, OsloMet – Oslo Metropolitan University, P.O. Box. 4, St. Olavs plass, N-0130 Oslo, Norway; 2 LEFO, Institute for studies of the medical profession, Oslo, Norway

**Keywords:** Medically unexplained symptoms, Primary care, Clinical knowledge and experience, Medical models, Framing

## Abstract

**Background:**

Medically unexplained symptoms (MUS) are a common yet challenging encounter in primary care. The aim of this study was to explore how general practitioners (GPs) understand and handle MUS.

**Methods:**

Three focus group interviews were conducted with a total of 23 GPs. Participants with varied clinical experience were purposively recruited. The data were analysed thematically, using the concept of framing as an analytical lens.

**Results:**

The GPs alternated between a biomedical frame, centred on disease, and a biopsychosocial frame, centred on the sick person. Each frame shaped the GPs’ understanding and handling of MUS. The biomedical frame emphasised the lack of objective evidence, problematized subjective patient testimony, and manifested feelings of uncertainty, doubt and powerlessness. This in turn complicated patient handling. In contrast, the biopsychosocial frame emphasised clinical experience, turned patient testimony into a valuable source of information, and manifested feelings of confidence and competence. This in turn made them feel empowered. The GPs with the least experience relied more on the biomedical frame, whereas their more seasoned seniors relied mostly on the biopsychosocial frame.

**Conclusion:**

The biopsychosocial frame helps GPs to understand and handle MUS better than the biomedical frame does. Medical students should spend more time learning biopsychosocial medicine, and to integrate the clinical knowledge of their peers with their own.

**Electronic supplementary material:**

The online version of this article (10.1186/s12875-018-0745-2) contains supplementary material, which is available to authorized users.

## Background

Medically unexplained symptoms (MUS) is an umbrella term used to refer to various symptoms that ‘have no identified organic basis’ [1], and ‘for which no adequate medical explanation can be found after a proper medical examination’ [2]. As such, MUS force general practitioners (GPs) to base clinical judgements on something other than biomedical evidence [3]. Cases involving MUS are said to ‘test the credibility of the doctor (…) for his or her inability to label the patient’s complaint’ [4], and it is well documented that MUS can be a challenge to both patient and doctor [5–7]. Those difficulties notwithstanding, MUS are among the largest categories of complaints in primary health care [8, 9]. In a recent Danish study, almost one in three patients belonged to this category [10]. Consequently, GPs need to understand and handle these patients’ complaints. Yet, not enough is known about how GPs actually do this. In this article, we therefore explore GPs’ approaches to understanding and handling MUS.

We use the concept of *frame* to explore GPs’ approaches to MUS. Frames are shared ways of ‘organising experience’ [11, 12]. Each complaint can be interpreted under different framings, and each frame indicates different approaches to patient management. Studies suggest that whereas patients expect or demand that GPs employ a biomedical frame, GPs prefer a biopsychosocial frame [13–17]. This is perhaps not surprising, as the biopsychosocial model is at home in primary health care. Yet other studies suggest the opposite [18, 19]; patients want support and compassion, but GPs provide somatic screening and intervention. Either way, the literature indicates a *tension* between a biomedical frame centred on disease and a biopsychosocial frame centred on the sick person [20]. This tension is heightened by health insurance policies and welfare bureaucracies that favour biomedicine [21, 22]. Little is known about how GPs negotiate those tensions, or how choice of frame affects patient management. This paper therefore explores how medical frames organise GPs’ understanding of MUS, and how this enables (or disables) patient management. To that end, we conducted focus group interviews with GPs about MUS.

## Methods

### Design, setting and participants

Three focus group (FG) interviews were conducted in Norway in the first quarter of 2015. The number of groups was considered appropriate for an explorative study. Recruitment took advantage of established groups in the continuing medical education program (see Table 1 for group characteristics). In Norway, there is a five-year specialization program to become a specialist GP, which includes regular group supervision. The groups were informed about the study beforehand, and none refused to participate. We purposively sampled groups with varied experience [23], in terms of years and place of practice. FG1 mainly included non-specialists in training, most of whom work in suburbs around Oslo; FG2 was a mixture of doctors in training and experienced specialists in general practice, most of whom work in rural areas in the east of Norway; FG3 included experienced specialists, most of whom work in Oslo. The interviews were audio recorded and lasted for 90–120 min.

**Table 1:** Focus group composition and participant characteristics

	Experience (yrs.)			Specialist (yrs.)			Age (yrs.)			Gender	
	< 5	5–10	10 >	Not	< 5	5 >	< 40	40–50	50 >	F	M
FG1	7	1	1	8	–	1	6	3	–	4	5
FG2	1	3	5	2	2	5	1	3	5	4	5
FG3	–	3	2	–	3	2	–	5	–	4	1

FGs are ‘artificially set up situations’ [24], ‘created and managed by the researcher’ [25], where participants and researchers *co-construct* [26] the data. It is therefore important to clarify researcher contributions to the data [27, 28]. EBR is a sociologist, KIR is a medical doctor trained in occupational medicine. EBR moderated the three interviews, KIR assisted. The semi-structured interview guide centred on experience with MUS and patient management (see Additional file 1). We asked about their experience with MUS, about what they considered typical features of patients with MUS, about what one should or should not do, and why. Moreover, we asked about the distinction between diseases and non-diseases, and about what diagnoses they used and why. We treated ‘MUS’ mainly as *placeholder* for conditions for which there are no biomedical evidence, meaning that apart from that criterion, we did not specify what conditions to discuss: we wanted them to decide. However, we did ask specific questions about sick listing, and in doing so, we implicitly excluded retired patients with MUS or patients with MUS who were already on permanent disability benefits.

FGs are good for producing concentrated amounts of data about issues for which it would be difficult to gather large sets of observations [25]. Additionally, by having groups of GPs engage each other in debate, FG methodology allowed us to use their experiences and perspectives as tools for exploration; they could give informed responses and rebuttals in ways we could not. Allowing participants’ responses to each other to drive the interviews was also a fruitful way of exploring those aspects we did not know in advance to look for.

### Ethics, consent and permissions

The Norwegian Social Science Data Services approved the study (project number 41259). All participants gave written consent to participating, and for using the data in publications. Participants were also given the option to check the data used for publication.

### Analysis

EBR transcribed in NVivo, drawing on Barbour's [29] annotation style. Italic font indicates emphasis; added information is in parentheses; brackets are used to describe events instead of representing them verbatim (typically non-lexical utterances, e.g. ‘[mhm]’); three stops indicate pauses in speaking (‘…’) or breaks in quotation (‘(…)’). All quoted excerpts were translated by EBR.

Our disciplinary backgrounds allowed us to combine methodological skill with analytical sensitivity informed by clinical experience. Although sense making was an analytical interest from the outset, our interest specifically in clinical experience and medical frames grew out of interpretative engagement with the data and the literature. After initial analysis and coding done separately by EBR and KIR, we discussed and decided on a strategy for further analysis. EBR analysed the data thematically, broadly in line with Braun and Clarke [30], combining *descriptive* and in vivo coding styles [31]. The final analysis made sense of the various ways the GPs understood and handled MUS in our data. The two main themes are presented as medical frames in the following section.

## Results

When discussing MUS, our participants alternated between 1) a biomedical frame, centred on disease, and 2) a biopsychosocial frame, centred on the sick person. Each frame accentuated different aspects of MUS. In the following, we describe how each frame organises GPs’ understanding of MUS, and how this affects them, and their approach to handling patients.

### MUS in the biomedical frame

The biomedical frame accentuated what is missing in MUS (objective signs of disease), and problems thought to flow from this absence. Consider excerpt A from FG1:

#### GP1

[T]here are rarely any specific issues with subjective *complaints*. That’s definitely what I find the most difficult [mhm]. What the patient says and feels, that’s what you have to deal with. And it’s very difficult to assess, say, pain, objectively. Or to assess … sadness, objectively [yes], anxiety, worries. So really, we’re in a situation where we have to *listen* to the patient, and perhaps sick list based on that. And, when the law says that (…) we have the *opportunity* to sick list, even when we cannot *point* to anything *specific*. Then we have no choice but to trust the patient. And, of course, in principle, the patient decides what he or she wants to *say*. And then that can be entirely correct, or it could be entirely wrong [in-breath yes]. But often it’s somewhere in between. Those are the difficult sick listing cases, definitely [mhm] ….

#### GP2

I think it’s difficult too, with regards to the legislature. Because it clearly *states* that there should be a ‘disease, injury or defect’ (a legislative paraphrase) [mhm]. Usually, it’s more of a borderline issue [mhm] (…).

#### GP3

And some of those sick notes are usually not the ones that last two- or three days. It’s the ones that are a bit longer that are difficult, when it comes to unclear symptom constellations, or how to put it? I think *that’s* where you’re *dependent* on what the patient *says* (…).

#### GP2

There are many difficulties with the whole issue of fatigue. Examined, and yet we can’t find anything, and then there are often a lot of burdens in their lives, which leads to the fatigue. And what are we to *do* about it [mhm]. Because, to sick list them … I mean, there’s no *disease* [mhm]. The way I see it.

#### GP3

Mhm. Tremendously difficult. (FG1).

The excerpt exhibits what was typical and related features of the biomedical framing of MUS. First, the focus throughout is on *the lack of objective evidence*. Thus, according to the GPs, ‘there are rarely any specific issues’ with MUS, GPs ‘can’t find anything’, possibly because ‘there’s no disease’ to be found (all from excerpt A). Some also pointed to the lack of scientific knowledge and explanation. For instance, one regretted not having ‘an explanation for these conditions (MUS) in medical science’ (FG2). When employing the biomedical frame, GPs thus understood and defined MUS negatively, in contrast with “normal” conditions for which evidence is obtainable and medical science has explanations on offer.

A second feature, and related to the former, is the strong emphasis on *subjective testimony as a problem*. Without objective evidence, GPs ‘have no choice but to trust the patient’ (GP1 excerpt A), i.e. they are ‘*dependent* on what the patient *says*’ (GP3 excerpt A). Having to trust the patient was unpopular, as it involved the risk of being misinformed or even deceived. Patient testimony was thus framed as unreliable: it could be ‘entirely correct, or it could be entirely wrong’ (GP1 excerpt A). In other words, subjective testimony was considered a problematic source of knowledge about patients’ conditions. Health insurance policy stipulates that impairment should have disease as its primary cause. Without evidence, the plausible presence of disease must be determined based on testimony. In the biomedical frame, sick listing thus becomes a problem of trust, and this is why some GPs felt it difficult to act responsibly as gatekeepers (see excerpt A).

Third, related to both lacking evidence and the low epistemic value attributed to testimony were frequent references to *negative feelings*, such as uncertainty and doubt. Some physicians were afraid that the patient might have a serious undetected problem, as expressed by a participant in FG2: ‘Perhaps there’s something else that I haven’t seen?’ Others emphasised how inability to obtain evidence spawned feelings of uncertainty, doubt and powerlessness. Consider excerpt B:

(…) we start to doubt how sick the patient is. Because we can’t quite objectively *grab a hold* of these things. We can’t do any blood tests, we can’t scan them or anything. And then we begin to doubt a little. (FG2).

The GP explicitly ties his doubt to the inability to ‘objectively grab a hold’ of MUS. It is because he ‘can’t do any blood tests’ or the likes that he begins ‘to doubt how sick the patient is’. It is noteworthy that lack of evidence results in doubts in patients rather than doubt in medical knowledge. Some voiced suspicion of malingering. For instance, a participant in FG3 talked about two cases concerning young men with back pains. She ‘couldn’t find anything wrong’ with their backs and concluded that they were unhappy with their jobs and wanted sick notes for their ‘supposed back pains’. Some also complained about feeling powerless. Consider excerpt C:

I can urge, or give medical *counsel*, and I can *suggest* that we try and up the workload in accordance with what is considered medically appropriate. But, in the end, when she says ‘No, I actually *cannot* work more (…) I have no choice but to *trust* the patient, and I really feel forced into doing what she wants [in-breath yes, mhm]. (FG1).

We emphasise that GPs *feel* powerless; their powers are no more restricted here than they are with biologically verifiable diseases like hyperthyroidism (legally, GPs cannot *make* patients do anything – they must counsel). But with MUS, GPs *feel* inhibited. Note also that the participant believes the patient to be healthier than the patient does. The GP’s problem, then, is the lack of authoritative warrants. Without evidence to back him, he feels that he cannot (or should not) force or sway the patient.

The biomedical frame thus accentuated the lack of objective evidence, the problem of trust and subjective testimony, and various troubling emotions. For those reasons, the frame also brought up frequent references to how MUS made GPs’ work difficult. Because the symptoms are ‘difficult to assess’, sick listing becomes difficult (‘what are we to do about it?’), elevating the risk of going into what one GP called ‘a stalemate’, i.e. an unfruitful therapeutic situation (FG1).

### MUS in the biopsychosocial frame

In contrast to the biomedical frame, the biopsychosocial frame accentuated what is present, and opportunities that flow from this presence. Thus, when talking about MUS in the biopsychosocial frame, the GPs emphasised understanding, confidence and competence. Consider excerpt D:

- A *nice* aspect of being a GP is getting to know people over time. And I’m thinking of my patient list a bit like my flock. I‘m looking out for them, over time, to get the most out of it. They’re going to be as comfortable as possible, so they can go to work, make money, pay taxes. And that means that you get to know people, and you can tell ‘Will it pay off to invest in a small sick note? Be a little proactive about it [mhm]?’ So that they’ll return to work *quicker*? Almost like a preventive measure [mhm]. And I do have quite a few ‘good girls’ and a few ‘good boys’, who will at times stretch the rubber band a bit too far [M: mhm]. And then, some people need a little sick leave. So you’ve got to be watchful (…). (FG3).

Excerpt D exhibits several prominent accents of the biopsychosocial frame. First, the participant expresses an *understanding* of the condition of patients he characterises as ‘good girls’ and ‘good boys’ – Norwegian slang for dutiful persons who tend to exert themselves too far. He also explains the condition by way of metaphor, saying such patients ‘will at times stretch the rubber band a bit too far’ – i.e. the body’s ability to recuperate (elasticity), is lost. In other words, he (feels that he) knows what is troubling his patient. Other patient types were suggested, such as ‘the double-labouring woman’, ‘between 37 and 43 years old, with three kids (…) and a job in the care services’, whose conditions were understandable to the participants: ‘It’s in the *entire* system, the *entire* body, and the burden becomes *too heavy*’ (FG2). This was typical of the biopsychosocial frame: MUS were discussed in terms of patient types the participants understood and could accept.

Second, because the participant in excerpt D feels confident that he understands, he also seems *confident about how to handle* these patients. He ‘can tell’ when a brief sick leave ‘will pay off’, and so he is ‘watchful’. In other words, he (believes that he) knows what to do. As a result, he does not seem worried about sick listing patients with MUS. In his experience with these patients, using sick notes for the present condition can work ‘like a preventive measure’ for a later longer, and possibly irreversible illness trajectory. Understanding MUS in terms of patient types thus seems clinically efficacious. The contrast with the biomedical framing of MUS in this regard is striking.

Third, the participant in excerpt D ties his understanding with his *clinical experience*: it is because he gets ‘to know people over time’ that he ‘can tell’ what is wrong. This, we suggest, is a seminal effect of the biopsychosocial frame: it invites GPs to draw on their clinical experience to make sense of MUS. It is not simply that GPs come to trust what their patients say. By drawing on their extensive clinical experience, GPs can acquire a holistic understanding of the sick person, enabling them to act with confidence. Note that ‘understanding’ does not imply veracity – the GP could have the wrong idea. What is implied is rather that the patient’s complaint is rendered meaningful in a clinically helpful way. Moreover, because GPs get ‘to know people over time’, trust is not (as much of) an issue. The credibility of patients’ suffering is not called into question. Consider excerpt E:

#### GP1

You have to *see* them over *time*, you have to get to *know* people, so you can *sense*-, or *form* a picture, over time. Is it *real*? Do they *have* these troubles, these impairments they claim to have? That you don’t have instruments to measure. And I’m thinking *this* is where being a doctor is *exciting* [Yes]! This is where the art of medicine comes in! And where people knowledge comes in! Whereas with these other conditions, if a leg is *broken* or you’ve *seen* a heart attack on EKG. *Alright* then (inaudible, chuckles) that’s technique. But its not much of an *art of medicine.* (…).

#### GP2

I think that when you *know* the patient, like (GP1) says, over a quite *extended* period of time, I think most of us would agree that … the suffering is *there*. I think one *feels* that quite well, that there is no *doubt* that these patients suffer, and are *sick*. (FG2).

The emphasis on clinical experience (‘people knowledge’) and ‘the art of medicine’ was at times coupled with a distancing from scientific medicine and medical training, as in excerpt F:

- (…) I feel that, in the course of an ordinary day, I can see *rather* a *lot* of patients, without having to use what I learnt in medical school, like *academic* or *scientific* (training). It’s more like … ‘yes, mhm, yes I understand, mhm’ (pretending to answer a patient). I mean, *that’s* what we spend our time doing. (FG1).

Thus, in the biopsychosocial frame, MUS concerns what they do know, instead of what they do not. Rather than worrying about the lack of objective signs of disease and evidence based treatment, talking about MUS in the biopsychosocial frame meant relying on clinical experience (with individual patients and patient types), informal explanatory models and interpretation. In the biopsychosocial frame, MUS thus become (more) tangible.

### Differences between groups

The use of frames differed across the focus groups. The group with specialists in training (FG1) relied heavily (though not entirely) on the biomedical frame. In contrast, the group of experienced specialists (FG3) relied almost exclusively on the biopsychosocial frame for discussing MUS. FG2, the group with the most variation in clinical experience, slightly emphasised the biopsychosocial frame. Similarly, outspoken preference for biomarkers (‘the more objective (…) the more we like it’) was frequently expressed in FG1 (the juniors), less frequently in FG2 (mixed group), and not once in FG3 (the seniors). Moreover, expressions of insecurity and frustration regarding patient management was frequently expressed in FG1, in contrast to FG3, whose members seemed confident about themselves and their own judgement. When the seniors in FG3 and FG2 voiced their frustration, it typically concerned bureaucrats and consulting physicians who did not accept the GPs’ clinical judgement and instead instigated ‘the burden of evidence’ on them.

## Discussion

Our analysis has shown how two medical frames shaped GPs’ understanding of MUS, and how this affected them and their approach to handling patients. Biomedical framing emphasised what is missing (objective evidence), made what is present (patient testimony) problematic, and manifested feelings of uncertainty, doubt and powerlessness. By comparison, biopsychosocial framing seemed to lessen and even solve some of those problems. In particular, it made the conditions understandable and turned patient testimony into a valuable source of information, which in turn made GPs more comfortable and confident. A main reason for these differences, we suggest, is that whereas the biomedical frame invites GPs to draw on formal and scientific knowledge (of little use with MUS), the biopsychosocial frame invites GPs to draw on their clinical experience to make sense of their patients’ problems. This enables them to make clinically efficacious distinctions between patients with MUS that give direction to clinical judgement.

In terms of patient handling, biomedical framing centred on what the patient has (disease or not). Since this is precisely what cannot be biomedically determined, handling (such as sick listing) became problematic. In contrast, biopsychosocial framing centred on how to improve the patient’s condition. For instance, the GPs suggested that short-term sick listing can alleviate stress and prevent long-term absence from work, and that being compassionate and supportive can help patients cope with their situation. Paraphrasing Stone [32], the biomedical frame thus manifested “the botanist”, bent on scientific classification, whereas the biopsychosocial frame manifested “the gardener”, bent on nurturing and making things “grow”. In terms of handling MUS, the latter mode currently seems more appropriate and effective.

Finally, the GPs with the most experience tended mostly to employ the biopsychosocial frame, whereas those with the least experience tended to rely more on the biomedical frame.

### Choosing medical frames

The biopsychosocial model is at home in primary health care, and seems better suited for handling MUS. So why was biomedical framing a prominent feature in the FGs? In short, because framing is not simply a matter of personal choice. For one, GPs’ framing practices are subject to external pressure: there is a strong institutional emphasis on the biomedical model of disease. Formally speaking, health related benefits are contingent on a biomedical account [21, 22]. When trying to secure disability pension for patients whom they consider sufficiently impaired, GPs therefore bear ‘a burden of evidence’, as one participant put it (FG2). Moreover, there is a strong cultural preference for clear-cut biomedical diseases and diagnoses in medicine [33–35]. As one participant put it, ‘The more objective the findings, the more we like it, because that means we can verify it [mhm]. What we *don’t* like are conditions where you have *zero* objective findings (…)’ (FG1). GPs are thus part of a culture that values objective evidence and unambiguous disease (and this preference is not restricted to medicine [36]). There is thus an impetus towards a biomedical framing of MUS. As one participant said, ‘we have to try to *create* this “cause effect” model that we should feel makes sense *ourselves*, that the patient should feel makes sense and that NAV (the national insurance bureaucracy) should feel makes sense’ (FG2). For these reasons, framing is not simply a matter of choice.

### Situating our findings

Our study is small, and the findings cannot be generalized to all GPs. However, although few studies have explored the effect of medical frames, our findings and their theoretical underpinnings are supported by – and shed light on tensions within – existing research literature.

First, regarding the negative effects of biomedical framing of MUS, studies typically report that GPs experience negative emotions when working with MUS, such as uncertainty, fear, frustration and powerlessness [2, 3, 37–41]. Yet few studies attempt to understand the cause of the negative emotions on a deeper level. We suggest viewing GPs’ emotions as frame related, as expressions of whether or not a frame promotes action and understanding. On examining the data presented in these studies, this interpretation makes sense. For instance, a doctor in one study by Warner et al. said MUS are challenging because ‘it doesn’t fit the medical mould (…)’ [38]. Our finding that GPs’ biomedical framing makes subjective testimony problematic could also help explain why patients are reported to feel distrusted and misunderstood, and that they must struggle to be recognised as legitimate sufferers [1, 16, 42–45]. Although others have pointed to the lack of fit between biomedicine and MUS [46, 47], we have not found studies that show *how* biomedical framing makes MUS problematic. Our findings thus help tie frequently reported problems of MUS to the biomedical model of disease: it is against such a background that trust in patient testimony, and lacking objective evidence and scientific explanations, become problematic.

Second, our finding regarding the positive effects of biopsychosocial framing also finds support in the literature. Although rarely highlighted, several studies reporting negative emotions also show examples of GPs feeling confident about their ability to understand and handle MUS. And typically, this is when they depart from a biomedical frame. For instance, Wileman et al. [39] reports that despite the GPs’ negative feelings, they ‘felt that showing an empathy with the patient, and taking an interest in them (…), enabled the patient to gain personal trust in the doctor’. Moreover, the GPs ‘felt they had the opportunity to ‘know’ such patients better (than other doctors), and build a relationship upon which successful management could be based’ [39]. GPs are also typically reported to explain MUS by considering the sick person in his or her psychosocial context [2, 3, 37, 39, 40, 48–50]. While the link between this understanding and a form of biopsychosocial framing is rarely explicated, it is certainly indicated. Moreover, studies of sick listing MUS in primary care indicate the need to assess patients’ complaints holistically, and emphasise the importance of trust and knowing the patient over time [49, 51, 52]. Finally, studies into occupational medicine support our claim that the epistemic valuation of patient testimony is frame related [12, 22]. In particular, Dodier’s description of the “clinical frame” and the “solicitude frame” resemble our description of biomedical and biopsychosocial frames, respectively: in the latter, ‘the patient’s complaints have the status of an ‘unconditional force’ and their legitimacy is not therefore called into question’ [12].

The work of Mik-Meyer [3, 49] approximates ours. She too finds that ‘biomedical classification and diagnostic tools (…) were replaced with trust and confidence when doctors were working with patients with MUS’ [3]. Yet Mik-Meyer claims that ‘MUS create an important, new role for doctors’, in which they ‘are encouraged to make judgements on the basis on something other than purely objective medical findings (…)’ [3]. We think that lacking objective evidence is an inherent part of clinical work. Instead of a new role, we suggest, what is required is the role belonging to what Jewson called “Bedside Medicine” [53], centred on the ‘total psychosomatic disturbance’ of the sick person. In other words, what is needed is a proper ‘general physician’ [54] (coupled, of course, with proper scientific research into the nature and causes of MUS).

Third, regarding our finding that the junior GPs relied the most on biomedical framing and expressed more insecurity and frustration than the seniors, the literature indicates that this is not coincidental. Studies suggest that understanding and handling MUS is more problematic for inexperienced GPs [41, 50, 55, 56]. Some indicate that ‘physicians who are in practice longer experience less stress from uncertainty than those in practice for shorter periods of time’ [57, 58]. Others report that junior GPs feel unsure of themselves specifically because of their lack of training and experience with MUS [41, 55], and are reported to be *less strict* gatekeepers than their experienced peers [59]. One possible reason for these findings is a selection mechanism, whereby those who are “biomedically minded” and insecure change job, whereas those who are biopsychosocially minded and comfortable stay. An alternative and likely complementary reason is that since biopsychosocial framing invites GPs to draw on their experience, there is a reciprocal relationship, wherein experience supports the frame, and the frame supports the use and generation of relevant experience. In other words, *biopsychosocial practice builds confidence*. More research is needed, and in that regard we note that experience is not limited to number of years – the type of experience (e.g. feeling that you succeed) likely matters most.

## Conclusion

We suggest that biopsychosocial framing, combined with clinical experience, enables GPs to understand and handle MUS better than biomedical framing does. However, that does not necessarily imply that biopsychosocially minded GPs benefit patients and society. Although similarities between MUS have been found [54], it remains a differentiated patient group, and there are few widely acknowledged efficacy studies (even the cautious indications of PACE are now in question, see [60]). Studies indicate that many (but not all) patients want more support, compassion and understanding [18, 19], and for GPs to be attentive to their personal circumstance [61]. (Note that there is no contradiction between these wishes and believing that one’s condition is rooted in an undetected somatic pathogen.) This supports the notion that biopsychosocial framing benefits patients as well as doctors. Moreover, fewer rounds of diagnostic screening and referral would save time and costs, which could benefit other patients. But there are also possible problems: biopsychosocial framing likely increases the tendency to medicalise ordinary troubles [62–64], and there is ample room for implicit bias [65, 66] in the clinical judgement of practitioners who are overly confident in their “people knowledge”. Clearly, more research is needed.

### Strengths and limitations

The strength of qualitative studies, such as focus-group interviews, is their ability to provide experience-based knowledge and insight, rather than a quantitative ranking of importance or the proportional distribution of opinions [25, 67]. Including doctors with different lengths of experience, specialists as well as physicians in training and doctors of both genders, ensures a diversity in experience, although we cannot draw robust conclusions. The inter-disciplinary collaboration between a sociologist (EBR) and a medical doctor (KIR) has potentiated critical reflection when interpreting the data. This can result in a more nuanced and balanced discussion, but in some cases also lead to less clear-cut conclusions than if only one perspective had prevailed. External validity or transferability can be assessed in relation to how the data are discussed [27]. As we show, our results are in line with previous research in this field. Moreover, we have presented our findings at medical and sociological conferences, and our conclusions were recognizable and credible to these different groups.

### Implications

If biopsychosocial thinking and clinical experience are central to GPs’ understanding and handling of MUS, than this should be reflected in research, teaching and practice. What is needed is an emphasis on the role of *clinical knowledge* [68, 69]. Clinical knowledge emerges in the course of practice, i.e. the daily chore of interpreting and interacting with patients, and applying general concepts to individual persons [68, 70, 71]. It is thus local, hermeneutic and experience based; its genesis bottom-up, contrasting top-down scientific and evidence-based knowledge. Such knowledge is the core of clinical reasoning and judgement [69, 71–73]. Yet not enough is known about its content and consequences [68, 74–76]. Thus, experienced-based ways of knowing must be studied further, so that they may be shared and scrutinised for the betterment of patients and practitioners [68]. In line with this, medical students should spend more time learning to think biopsychosocially, and to integrate the clinical knowledge of their peers and seniors with their own. There is no denying the success of the biomedical model, but its uses are limited: quality primary care is impossible without acknowledging that personality and circumstance are major constituents of patients’ health [77, 78].

## Additional files

Additional file 1: Translated interview guide. English translation of the semi-structured interview guide. (DOCX 13 kb)

Additional file 2: The OPR reports. (ZIP 65 kb)

## Abbreviations

FG: focus group
GP: general practitioner
MUS: medically unexplained symptoms
