# Supplementary material for: How general practitioners understand and handle medically unexplained symptoms: a focus group study
Source: BMC Fam Pract. 2018 May 2;19:50. doi: 10.1186/s12875-018-0745-2 (PMC5932817; doi:10.1186/s12875-018-0745-2)
Supplement: Supplementary file 1 — Translated interview guide. English translation of the semi-structured interview guide. (DOCX 13 kb) [file 12875_2018_745_MOESM1_ESM.docx]

1. Opening questions
   1. Specialist or in training
   2. Years of experience as GP
   3. Place of practice
   4. Other practical experience working in the health services
2. MUS or uncertain illness
   1. Some doctors say that questions of sickness and sickness certification are challenging when handling patients with MUS. Consider the following statement (by GP from an online and public forum for general practice):
      1. “Some patients are in obvious good health, while others are obviously very sick. The problem arises when we operate in the so-called “grey area”: fibromyalgia, whiplash, chronic fatigue syndrome, personality disorder, and chronified bullying by employers. A single case can utterly drain a GP with a certain level of commitment”.
      2. What are your thoughts? Is this a recognisable way to think about MUS?
3. Patient types
   1. Have you ever had a patient with what one might call MUS or uncertain illness?
      1. Describe a typical patient
      2. What characterises them?
      3. Could you give an example?
   2. How do you approach such patients?
      1. What is important, and what should you not do?
   3. What is it like to work with these patients?
4. Diagnoses
   1. How do you decide what diagnosis to use?
   2. Some doctors are sceptical or negative about certain diagnoses, e.g. ME or fibromyalgia. Why do you think that is? What are your thoughts?
   3. Do you use such diagnoses?
5. Sickness certification and work capability assessment
   1. When is sickness certification appropriate? When is it not?
6. Referral
   1. To whom do you refer?
   2. What are your experiences with regards to referrals? Are some specialists easy to cooperate with? Or hard?
7. Health insurance
   1. How is your cooperation with NAV (the national insurance bureaucracy)?
