# Supplementary material for: How general practitioners understand and handle medically unexplained symptoms: a focus group study
Source: BMC Fam Pract. 2018 May 2;19:50. doi: 10.1186/s12875-018-0745-2 (PMC5932817; doi:10.1186/s12875-018-0745-2)
Supplement: Supplementary file 2 — The OPR reports. (ZIP 65 kb) [file 12875_2018_745_MOESM2_ESM.zip › Cover letter round two.docx]

**Dear Editor,**

Thank you for the chance to improve our manuscript. Based on your comments, we are now pleased to resubmit a revised manuscript for consideration for publication in *BMC Family Practice*. Below you will find our detailed responses to your comments, including descriptions of any changes made to the manuscript. All revisions in the manuscript are made using ‘track changes’.

**Remark 1**: “The first sentence in the Background is in my opinion not necessary as you describe the aim of the article at the end of the Background which I think is appropriate”

*Our response*: Thank you for this suggestion. We have removed the first sentence, and made the necessary alterations to accommodate for that in the following sentences. The opening to the background section now reads, “Medically unexplained symptoms (MUS) is an umbrella term used to refer to various symptoms that ‘have no identified organic basis’ [1], and ‘for which no adequate medical explanation can be found after a proper medical examination’ [2]. As such, MUS force general practitioners (GPs) to base clinical judgements on something other than biomedical evidence [3]” (P2: L7-11).

**Remark 2**: “I do think that it is important to state whether the saturation was reached? Please, explain in the Methods. This is in my opinion an important information”

*Our response*: Thank you for this chance to clarify. We understand your remark as a question about whether our analysis reached a point where we felt that we had learned what we could from our data, and sufficiently so in order to answer our research question. This is what is often meant by saturation, and if so, the response is yes; our analysis did reach that point. As such, we could talk about having reached saturation. To that effect, we have added the following two sentences to the methods section: “The final analysis made sense of the various ways the GPs understood and handled MUS in our data. The two main themes are presented as medical frames in the following section” (P4: L26-28). So to clarify, what we have done amounts to saturation, in the sense we think you are using that term. However, we hope you will accept our reasons for not using the term ourselves, which are two-fold:

- Firstly, saturation originates from – and is tied to – a specific approach to qualitative analysis called Grounded Theory (GT). In GT, its meaning is more specific, denoted by the full name “theoretical saturation”, because the aim of GT is to generate field sensitive theory, and the theory should be saturated with data from the field. There is much to learn from GT, but there are also some epistemological principles in GT that we and many others disagree with (cf. Malterud et al 2015). Since we have not done a GT analysis, we would prefer not to use concepts from GT.
- Second, the concept is often used in problematic ways – in particular outside the bounds of GT. More often than not, it is used to signal quality – but without really saying anything about what has been done in the analysis or how the verdict of “saturation” was reached. Moreover, it is no simple matter to distinguish between saturation as in having reached a full and complete description on the one hand, and saturation as in the researcher having lost her “appetite” for new insights and variations on the other (cf. O’Reilly and Parker 2012 for a critic of the uses of the saturation-term in research). Because of the ambiguity associated with problematic usage of the term, we see no communicative advantage to using it in a paper that does not follow GT.

For those reasons, we have opted not to talk about saturation in the manuscript. But to reiterate, we share the sound principle that any study a) should gather enough data, and b) analyse them thoroughly enough, to be able to answer the research question. And we believe we have done just that. We hope our response is acceptable.

**Remark 3**: “In the sentence: The number of groups was considered apt for an explorative study, please write the full word for "apt". I interpreted it as appropriate, but I am not sure if this is ok”

*Our response*: Thank you for noticing. Your interpretation was correct, and your comment was *appropriate*. Now, our spelling is too (P3: L6).

**Remark 4**: “In my opinion, the Results section should not have any references. The Results section should only report on your results and the discussion is a place for explaining of your results and comparing them to the findings of other studies. In fact, I think that the entire paragraph "Choosing medical frames" belongs to the Discussion section”

*Our response*: Thank you for your suggestion. We have placed the entire paragraph “Choosing medical frames” in the discussions section (P11: L1-16). We have also made some modifications to the introduction to that paragraph, in particular in light of remark 6 (see below), but also to help it sit well in its new place. We also made slight alterations to the rest of the paragraph, also to help the paragraph sit in its new place (P11: L4, 5 and 8).

**Remark 5**: “I would suggest that the Discussion is written in the past tense. I.e.: Our analysis showed...”

*Our response*: Thank you for this suggestion. We have made the appropriate alterations, so that references to the reviewed results are written in past tense (see P10: L6-23).

**Remark 6**: “I agree with the reviewer 2 about the comment on the biopsychosocial approach in family medicine. It is one of the main competences of the European definition of family medicine/general practice. So, family physicians should manage patients with a biopsychosocial approach and your findings actually prove that they do well. I think it is important that you emphasise that”

*Our response*: We agreed with reviewer 2 that the biopsychosocial approach is central to family medicine, but unfortunately, we were caught up in his assertion that it was against logic to talk about biomedicine. We have made two alterations to emphasise the role of the biopsychosocial model in family medicine. *First*, in the second paragraph of the Background section, we have inserted the following sentence: “This is perhaps not surprising, as the biopsychosocial model is at home in primary health care” (P2: L23). In context, it reads as follows: “Studies suggest that whereas patients expect or demand that GPs employ a biomedical frame, GPs prefer a biopsychosocial frame [13–17]. This is perhaps not surprising, as the biopsychosocial model is at home in primary health care. Yet other studies suggest the opposite [18,19]; patients want support and compassion, but GPs provide somatic screening and intervention” (P2: L21-25). The *second* alteration is in the introduction to the paragraph called “Choosing medical frames” (which has been moved to the Discussion section, see remark 4 above). We have inserted three new sentences, which read as follows: “The biopsychosocial model is at home in primary health care, and seems better suited for handling MUS. So why was biomedical framing a prominent feature in the FGs? In short, because framing is not simply a matter of personal choice” (P11: L2-4). Together, we think believe these alterations brings emphasis to the important role of biopsychosocial medicine in general practice.

***

In addition to these changes, we have changed the contact information for the corresponding author, to compensate for the new university status (see P1: L7-8). This was forgotten in the last round, where we only remember to change the name of the institution.

REFERENCES:

Malterud, K., V. D. Siersma, and A. D. Guassora. 2015. ‘Sample Size in Qualitative Interview Studies Guided by Information Power’. *Qualitative Health Research*, November, 1049732315617444. https://doi.org/10.1177/1049732315617444.

O’Reilly, M., and N. Parker. 2013. ‘“Unsatisfactory Saturation”: A Critical Exploration of the Notion of Saturated Sample Sizes in Qualitative Research’. *Qualitative Research* 13 (2): 190–97. https://doi.org/10.1177/1468794112446106.
