# Supplementary material for: How general practitioners understand and handle medically unexplained symptoms: a focus group study
Source: BMC Fam Pract. 2018 May 2;19:50. doi: 10.1186/s12875-018-0745-2 (PMC5932817; doi:10.1186/s12875-018-0745-2)
Supplement: Supplementary file 2 — The OPR reports. (ZIP 65 kb) [file 12875_2018_745_MOESM2_ESM.zip › Cover letter.docx]

**Dear Editor,**

Based on the comments from the reviewer and yourself, we are now pleased to resubmit our revised manuscript for consideration for publication in *BMC Family Practice*. Below you will find our detailed responses to the reviewers, including descriptions of any changes made to the manuscript. All revisions in the manuscript are made using ‘track changes’.

Before moving on to the responses and changes made, we hope you will forgive us for saying that we were somewhat surprised by some of the reviewers’ comments. It is our impression that reviewers’ reports should give constructive and reasoned comments, presented in a comprehensible manner. However, as you will see reflected in our responses, we often felt that the reports fell short of these basic standards. Many of the comments – in particular in the report from reviewer 1 – were difficult to understand the reason for, and did not give indications as to what kind of changes were suggested (such as “Methods section is to be modified”). Moreover, far too often we struggled to understand the reviewers’ intentions, due to bad English. Naturally, we have done our best to use the reports to improve our revised manuscript. Moreover, since we found several of the comments problematic, we are thorough in explaining why they were not taken into account.

If our revision and our responses are found to be less than satisfactory in any regard, we are willing to make further revisions. However, for the reasons stated above, if you should find the need for further reviewing, we ask that you not use reviewer 1.

**Response to reviewers**

Dear reviewers, thank you for your reports. To ensure that all of your remarks were answered we have quoted and replied to each in a stepwise manner. Please note that, unless we state otherwise, all references to pages and lines in this document refers to the revised manuscript with track changes. All changes made are described in a systematic and readable way.

Reviewer 1: Polona Selic

**Remark 1**: “The MS is too long”.

*Our* *response*: We have interpreted MS as a short form for manuscript, and hope that is correct? Without reference to a specific part that should be shortened, or any reasons why you think the manuscript is too long and/or why the length is problematic, we did not feel we could use this remark to make informed alterations to the manuscript.

**Remark 2**: “Methods section is to be modified and Data Analysis is to be described more thoroughly”.

*Our* *response*: Without any indication as to how you think the methods section “is to be modified“, we were unable to use this remark to make alterations to the methods section. Likewise, although we understand that you want the data analysis “to be described more thoroughly”, the remark is difficult to abide without any indication of what direction you would like us to take (there is more than one path to thoroughness). We note, however, that changes have been made to the methods section, including the description of the analytical procedure (see our responses to reviewer 2).

**Remark 3**: “Results section is also too long and above all not very clear. Please, use Miles MB, Huberman AM. Qualitative data analysis. Thousand Oaks: Sage, 1994. The categories and concepts are advised to be tested in the following steps: [for the purposes of writing this response letter, we leave out the six steps, and the suggested paper]”.

*Our* *response*: We cannot make informed changes to our manuscript based on your remark that the results section is too long. We would have had to know something about which parts are redundant, or, for instance, if you think everything should be kept but described more effectively. It is unfortunate that you did not find the results section to be very clear either. You ask us kindly to use Miles and Huberman’s approach, and redo our entire analysis based on that. Please note that we are familiar with their approach and have also read and considered the paper you suggested. However, you do not argue as to why this would be the preferred approach (as opposed to the one we have used, or any other approach for that matter). It is far from apparent that Miles and Huberman is a better choice, as there are more ways than one of doing qualitative research. Although we do not understand your reasons for asking us to use this approach, we do think it helps us understand why you find the results section long and unclear in the first place: If your preference is matrixes and tables, then our chosen style of reporting qualitative data is a poor match. But if the problem is different preferences of doing qualitative research, then we see no reason why we should abandon our current approach. We think our approach is sound and that our way of presenting the data is in line with good current qualitative research.

**Remark 4**: “In Discussion sections, authors should refer to results which shall be organized in tables”.

*Our* *response*: We agree that discussion sections should refer to the results, as do ours: We refer to each of our main results in the discussion (the negative consequences of biomedical framing, the positive consequences of biopsychosocial framing, and the relative distribution of framing according to experience in our small sample). However, we disagree that results “shall be organized in tables”. It is not readily apparent to us that they should. Many (if not most) qualitative studies report their findings without the use of tables. Without any arguments as to why you think results should be organised in tables, we do not feel compelled to alter our presentation of the results. In our view, the communication of results in qualitative research is best when the data is presented as part of the text body, with careful introductions and explanations surrounding each excerpt.

**Remark 5**: “References should be selected, authors are advised against using references older than 10 years”.

*Our* *response*: We thank you for your advice, but find it deeply problematic. If the purpose of referencing is to document the current lay of the land (which is not always the purpose), then yes, it seems reasonable to avoid using outdated research. But when is something outdated? Ten years may be short for some phenomena, such as the continental drift of tectonic plates or foundational insights into social science methodology, but rather long for other phenomena, such as the ranking of schools and universities, which is updated every year or so, or how active practitioners today think about MUS. Moreover, it is not always the date but the place that should call for critical assessment. Are, for instance, lessons learnt in surgeries in Norway relevant for, say Slovenia or the UK? We think so, but it is important to consider. Our most important reason for rejecting the advice, however, is what the sociologist Robert Merton called “the fallacy of the latest word” – i.e. the mislead preconception that what comes last is better than what came before. As we make clear, we think there are important lessons to remember from the age of bedside medicine, before the rise of modern hospitals. There are current uses of historical knowledge and insight, uses that would be lost were we to subscribe to the belief that 11 year old research is outdated. In sum, our paper engages with references spanning the latest up to data research about MUS in primary care and classic yet relevant papers and books. We see no fault in that, as long as each reference serves as purpose.

Reviewer 2: Vojislav Ivetić

**Remark 1**: “Page 2, Line 10: it is very important to completemente part about lack of definition for MUS with: New DSM - V classification, which use new term "somatic symptom disorder" and with possibilites to think about unrecognized psychiatric illness (panic, anxiety, depression, etc.),.. Add sentence or two on that subject”.

*Our* *response*: Thank you for this comment. We agree that, overall, there is much to be learned from considering the various ways the umbrella term MUS has been classified. However, we fail to see how an added mention of somatic symptom disorder (or central sensitivity disorder, bodily distress disorder, functional somatic syndromes, etc.) would help the overall argument in this manuscript. Moreover, the MUS term does not lack a definition – it is defined in the literature, precisely by the presence of symptoms on the one hand, and the lack of identified signs of disease and a widely held scientific explanation on the other. It could of course be added that the MUS term is used in a variety of ways in the medical literature (e.g. in terms of number of symptoms, severity, persistence, and behavioural features such as frequency of attendance), but we do not think this would help the reader understand our argument. Therefore, we opt not to add any sentences on that matter, and hope our decision makes sense in light of our arguments.

**Remark 2**: “Page 2, Line 16: There is many studies about "how GPs handle patients with MUS", for example: CVT (cognitive behavioral techniques), medicines, etc...Please improve that sentence with facts, or add another sentence about it !”.

*Our* *response*: Thank you for this remark. If we understand you correctly, you are saying that there are clinical guidelines and techniques available, and that the effects of these have been studied. We agree. However, what we are referring to here, are studies that seek to describe how practicing GPs in fact go about their work, and how they in fact understand patients with MUS. That is why we say, “not enough is known about how GPs *actually* do this” (P2, L16, original manuscript, emphasis added). We are not claiming that guidelines do not exist, but rather that we know too little about how MUS is understood and handled in actual practice. And when we say that “not enough is known” we are of course not denying that some things are known (we refer to many of the relevant studies in the discussion).

**Remark 3**: “Page 2, Line 21: The BASIS of the work of GP specialist is "biopsychosocial frame",.. that is the difference between Family Medicine (GP) specialisation and most of the others medical specialisations. So sentence "GPs prefer a biopsychosocial frame" is not logical, because that is basis of GPs training and work ! Please change / improve that sentence in contence describe / explained.”.

*Our* *response*: Thank you for this comment. We agree that the biopsychosocial model is at home in family medicine, and that this is reflected in the teaching. However, you seem to imply a sharper dividing line between family medicine and other branches of medicine than we think there are grounds for. GPs are taught in biomedicine as well, and – as others have shown – biomedicine is a seminal part of doctors professional identities, acquired through training (cf. *How doctors think* by Kathryn Montgomery). Moreover, biopsychosocial medicine is not restricted to family medicine – not in terms of teaching nor practice (nurses learn it too, for instance). Moreover, since the research we cite (se P2: L21-22) support our claim that GPs both rely on biomedical and biopsychosocial thinking, which legitimates our inquiry into the role of these models, we respectfully disagree that our description goes against logic.

**Remark 4**: “Page 2, Line 27-28: In sentence "Our aim, then, is to explore,.... ", it is not cleare: Do you take into account ONLY these two frames (any other by literature ? Third, fourth etc ? ) , and What did you thought about "patient management" (management considering sick leave only ? Or managament considering decrease of main symptoms ? ) Please explain and add sentence with explanation”.

*Our* *response*: Thank you for this question. A difficulty in reporting qualitative work is often that the research question changes throughout the analytical process. As we explained in the methods section, “our interest specifically in clinical experience and medical frames grew out of engaging with the data and the literature” (P4: L11-13, original manuscript). In other words, we did not initially start out this way, but ended up finding that the distinction between these two models (or frames) helped us make sense of our data. In order to make this clearer, we have changed the sentence to which you reacted. It now reads “This paper therefore explores how medical frames organise GPs’ understanding of MUS, and how this enables (or disables) patient management” (P2: 27-28). This is what the paper does, although it was not our specific aim from the outset. Thank you for helping us clear that up.

**Remark 5**: “Page 3, Line 3: What was criterion for only 3 focus groups ? Why not 4 or 5 for example ? Please explaine and add some sentence about it”.

*Our* *response*: Thank you for raising this question. We did three focus groups because, based on experience and the literature, a small number can suffice in explorative qualitative studies. Had we intended to learn something that could be generalised statistically, or had we subscribed to notions from grounded theory such as “saturation”, we would had gone for more interviews. However, as stated, our aims were explorative, to be able to raise interesting points for discussion and reflection. More to the point, we aimed to gather a varied set of experiences from working with MUS: not many experiences, but different experiences. For that reason, we wanted people with different experience in terms of how long they have worked, and where they work (big city, suburbia or rural). In order to accommodate your request, we have added one sentence to clarify, which reads: “The number of groups was considered apt for an explorative study” (P3: L3-4).

**Remark 6**: “Page 3, Line 3: FG were only in Oslo ? Or in other cities also ? Only in cities or also in rural areas ? Explaine and add some sentence about it.”.

*Our* *response*: Thank you for this chance to clarify. We have changed our descriptions of the groups to include the place they work. It now reads: “FG1 mainly included non-specialists in training, most of whom work in suburbs around Oslo; FG2 was a mixture of doctors in training and experienced specialists in general practice, most of whom work in rural areas in the east of Norway; FG3 included experienced specialists, most of whom work in Oslo” (P3: L9-12).

**Remark 7**: “Page 3, Line 4: What was criterion for recruintment of GPs i.e. for purposive sample ? Please explaine”.

*Our* *response*: Again, thank you for this reminder to clarify. We have added to the sentence about purposive sampling, which now reads: “We purposively sampled groups with varied experience [23], in terms of years and place of practice” (P3: L8-9).

**Remark 8**: “Page 3, Line 4: How many GPs was invatied ? How many refused to participates ? Please explaine and add text”.

*Our* *response*: As we explain, we recruited established groups via the continuing medical education program. That means that the groups existed beforehand. We asked three groups if we could come and chair their next session. The groups discussed and accepted our request. No one refused to participate. We have clarified this in the text with the following sentence: “The groups were informed about the study beforehand, and none refused to participate” (P3: L7-8).

**Remark 9**: “Page 3, Line 7: What is a main reason that FG3 was smaller then others, and include much more females (considering facts, that you use purposive group sample) ? Please, explaine and add text”.

*Our* *response*: As we explained above, the groups already existed. The reason why FG3 was smaller is that this group has fewer members. Because this relates to matters already clarified, we have not added any further text.

**Remark 10**: “Page 3, Line 9: Improve Table 1, with new data mentioned above”.

*Our* *response*: We have not added anything in the text that needs to go into the table. We hope you agree with our assessment.

**Remark 11**: “Page 3, Line 14: Is that sentence means that EBR and KIR were present all time on all FGs ? Please explaine”.

*Our* *response*: As stated, “EBR moderated the interviews, KIR assisted” (P3: L14, old manuscript). That of course required the presence of both of us. We see no reason to state that we did not leave during the interview (but we would have clarified it if we had!). It is, to the best of our knowledge, not conventional to state that one did not leave. But in order to minimize the chances of confusion, we have modified the abovementioned sentence so that it now reads: “EBR moderated the three interviews, KIR assisted” (P3: L18).

**Remark 12**: “Page 3, Line 15: You mention "semi structured interview guide",... Please add that guide as a part of research as Table 2, or Graph 1, etc... ”.

*Our* *response*: Thank you for this chance to clarify. Our original manuscript was thin with regards to the interview questions we asked. However, since the interview guide was not used much, we have chosen instead to provide a description of the sorts of questions we asked our groups, in a section that now reads as follows: “The semi-structured interview guide centred on experience with MUS and patient management. We asked about their experience with MUS, about what they considered typical features of patients with MUS, about what one should or should not do, and why. Moreover, we asked about the distinction between diseases and non-diseases, and about what diagnoses they used and why” (P3: L18-22).

**Remark 13**: “Page 3, Line 16: "... no biomedical evidence",... How about missed psychiatric diagnosis? All that diagnosis have thier own diagnosis criterias, without biomedical evidence (except central nervous system hormons level,... but that is not a routine GPs test to investigate,... ). Please change that sentence and explaine more...”.

*Our* *response*: As we clarify in the text, we use the term MUS as a “placeholder”, meaning that we are not asking them to think of any conditions specifically, other than that they share the defining feature of MUS – the presence of subjective symptoms and the absence of objective signs of disease. It was our intentions to have the GP talk about whatever conditions they wanted to discuss, as long as they included those defining features. So for instance, the topic of missed psychiatric diagnosis came up as a concern. But so did a lot of other things. In order to clarify, we have made some alterations, so the section now reads “We treated ‘MUS’ mainly as placeholder for conditions for which there are no biomedical evidence, meaning that apart from that criterion, we did not specify what conditions to discuss: we wanted them to decide” (P3: 22-24).

**Remark 14**: “Page 3, Line 16: "... by asking about sick listing,... " Is that means that you thought only about MUS patients who work (have job) ? How about MUS patients who are unemployed?? Please change and complement the sentence”.

*Our* *response*: We did not think only about patients who have a job. The point is that sick listing came up as a topic, which lead the discussions primarily in the direction of patients for which that is a relevant intervention – i.e. adult patients who could potentially be at work. But not just patients with a job – the discussions altered between describing patients inside and outside work life (not to mention those in between). The point was to be clear about how we steered the discussion, and why consequently our paper says little about children with MUS. In order to avoid confusion, we have altered the sentence. It now reads “However, we did ask specific questions about sick listing, and in doing so, we implicitly excluded retired patients with MUS or patients with MUS who were already on permanent disability benefits” (P3-4: L24-2).

**Remark 15**: “Page 3, Line 21: add reference for a "they could give infromed ,.... "”.

*Our* *response*: We see no need to refer to published research in order to support our claim that GPs are *informed* about family medicine as practice in ways that the authors – who are not GPs – are not. “Informed” does not mean that their insights are true or accurate: The claim is that they could speak in ways that are shaped by their practical experience in ways we could not.

**Remark 16**: “Page 4, Line 15: Did you obtained any measurable categories, from the codes you recognized ? If yes, please add to a text,.. if not, please explaine why not ?”.

*Our* *response*: We have tried to understand the precise meaning of your question, but it eludes us. We did not come across categories we saw fit to measure. Nor would we talk about “recognising” codes. We *create* codes; they are the fruits of our interpretative work. We think, therefore, that the answer to your question is no. We also fail to see why we should explain the fact that we did not “obtain measurable categories”. Why does it need explaining? We interpret your question as coming from a different philosophical position than ours – it makes sense to talk about “measurable categories” and “code-recognition” from a positivist tradition, but not from a traditional interpretivist or hermeneutical tradition – by far the norm in qualitative research. We have made slight changes to highlight our interpretative stance on P4: 15-16, in a sentence that now reads, “our interest specifically in clinical experience and medical frames grew out of interpretative engagement with the data and the literature”.

**Remark 17**: “Page 4, Line 22: "objective signs of disease,.. " ,.. Most of the MUS patients have symtpoms (only subjective, like for example: fatigue, pain, vertigo, etc) ! Very few of them signs (objective like: tahycardia, high blood preasure etc),.. So if you exclude symptoms (subjective) from the MUS patients, you exclude MOST of the MUS patients ! Please explain,..”.

*Our* *response*: We fail to catch your intended meaning. You seem to imply that we are excluding subjective symptoms from patients with MUS. Let us therefore emphasise: We are not making any claims or the sort you (seem to) imply. We are not excluding subjective symptoms, nor do we claim to. Our claim is that when employing a biomedical frame, the GPs were preoccupied with the missing evidence rather than with the present symptoms (and we use the verb “accentuate” to denote that it is a matter of degree). We therefore do not see what it is that we should explain. From what you write, it would appear that the basis of your comment is the fact that we use the phrase “objective signs of disease”, but we fail to see how usage of this phrase leads you to believe we are excluding subjective symptoms. As it stands, we can make no alterations to accommodate your remark.

**Remark 18**: “Page 4, Line 27: Again,.. what was the aim of the FG works ? Problems of the sick lists or decrease of symptoms with MUS patients ? Explain please. ”.

*Our* *response*: We fail to understand your intended meaning. The aim is to explore how GPs understand and handle MUS. We explored that by engaging GPs in focus group discussions about their work with MUS. The discussions took many turns, but questions of sick listing was a frequent topic. We have not indicated anywhere that the aim is “decrease of symptoms” (the phrase is never used) and therefore fail to see what it is that needs explaining. Again, if the fault lies with us, we apologise.

**Remark 19**: “Page 5, Line 19: "typically framed in the literature",.. please add reference ! ”.

*Our* *response*: The point about adding this sentence was to call attention to similarities between our GPs’ biomedical framing, and the way MUS is defined in the research literature. It was supposed to be a neat comment. And we thought that since we had already described how MUS is described in the opening section, with references and all, that that would be enough. However, since it does not appear to work according to our intentions, we have opted to strike the sentence entirely.

**Remark 20**: “Page 5, Line 28: Again same dilema,.. Sick listing vs. decrease of MUS symptoms,.. What was primary aim of discusion ? Please explain.”.

*Our* *response*: Again, as above (response to remark 18), we fail to see what the dilemma is. We have never used the phrase “decrease of symptoms” and further fail to understand your intended meaning. We therefore do not know how to go about explaining this dilemma. We did discuss sick listing, as we indicated in the methods section, but also disability pension, diagnostics, health insurance, health policies, referrals, therapeutic dialogues, doctor-patient relationships, and the boundary between diseases and non-diseases.

**Remark 21**: “Page 8, Line 7: Excerpt E,... From what FG was made ? Please add to text”.

*Our* *response*: Thank you for this comment. It is important to clarify from which groups each excerpt is drawn. However, we do clarify that excerpt E is from FG2 (at the end of the excerpt, as in the other excerpts, see P8: L15, original manuscript, P8: L20 in the revised manuscript).

**Remark 22**: “Page 9, Line 11: "The specialist in training group FG1,... ",.. Did you mean "GP trainees" ? Please explain and add in text! ”.

*Our* *response*: Thank you for this question: In Norway there is a 5-year program for specialization to become a GP. Thus in this context we mean GPs who are on their way to becoming specialists. As we realize that this differs between countries we have added an explanation of this in the methods section: "In Norway, there is a five-year specialization program to become a specialist GP, which includes regular group supervision" (P3: L5-7). We also took this opportunity to improve the sentence you reacted to, which now reads “The group with specialists in training (FG1) relied heavily (though not entirely) on the biomedical frame” (P9: L16-17).

**Remark 23**: “Page 10, Line 1: "invites GPs to draw on their clinical experience,... " GP specialist have their on specific skills like "communication skills", "CVT technique", "home visits" etc,..”.

*Our* *response*: We agree that GP specialists have various skills. But we fail to see the meaning of your comment: We appear to agree (?). The point we are trying to make is that biopsychosocial framing makes certain experiences (and indeed experience based skills) relevant and important in ways biomedical framing does not.

**Remark 24**: “Page 10, Line 8: Is there any references in literature, so you can compare that statment ? ”.

*Our* *response*: We assume you are referring to the following sentence: “For instance, short-term sick listing can alleviate stress and prevent long-term absence from work, and being compassionate and supportive can help patients cope with their situation” (P10: L7-8, original manuscript). That sentence is referring to what our participants said, and as we understand your comment, you are asking us to consider whether a reference in the literature can serve as a basis of comparison. But we fail to see clearly what sort of comparison you are looking for. A reasonable guess is that you are asking whether our GPs’ claims are supported by published research. If that is the case, we have two answers. The first is that we do not know of any relevant literature. We have not come across studies to test whether sick listing can alleviate stress and prevent long-term sickness absence specifically for patients who are not yet – but may become – sick. I.e., we know of no study of “pre-emptive sick-listing”. (There are several studies into the harmful effects of long-term sickness absence, but that is quite different). We further hope no one has found warrants for doing an RCT to uncover whether “being compassionate and supportive” can be beneficial. But the second and more important answer is that checking whether our informants are correct or not is beyond the scope of our paper.

**Remark 25**: “Page 11, Line 5: Add references for first and second sentence”.

*Our* *response*: In the third sentence, we do give a reference. We write (we include here the first and second sentence for clarity): “Although rarely highlighted, several studies reporting negative emotions also show examples of GPs feeling confident about their ability to understand and handle MUS. And typically, this is when they depart from a biomedical frame. For instance, Wileman et al. [39] reports that despite the GPs’ negative feelings, they ‘felt that showing an empathy with the patient, and taking an interest in them (…), enabled the patient to gain personal trust in the doctor’. Moreover, the GPs ‘felt they had the opportunity to ‘know’ such patients better (than other doctors), and build a relationship upon which successful management could be based’ [39].” Following that extended reference, we cite several other studies. We opted to do it this way, because it is unhelpful to cite a reference to validate a claim that something “is rarely highlighted”. How should the reader go about checking for something that is not highlighted? For that reason, we went for showing examples, to illustrate similarities in ways GPs in other studies are reported to talk about MUS, and how our GPs did it. We think this is clear and helpful to the reader. If, however, the editors disagree, we are of course willing to put some of the later references in brackets at the end of the first and second sentences too.

****

In addition to the changes detailed above, we have made one alteration in the institutional affiliation of the corresponding author EBR (P1: L4-5). The reason why is that what was a university college at the time of submission is now (since January 12^th^ 2018) a university (see <https://www.hioa.no/eng/HiOA-is-now-known-as-OsloMet-Oslo-Metropolitan-University>).
