# Supplementary material for: How general practitioners understand and handle medically unexplained symptoms: a focus group study
Source: BMC Fam Pract. 2018 May 2;19:50. doi: 10.1186/s12875-018-0745-2 (PMC5932817; doi:10.1186/s12875-018-0745-2)
Supplement: Supplementary file 2 — The OPR reports. (ZIP 65 kb) [file 12875_2018_745_MOESM2_ESM.zip › Cover_letter ORIGINAL.docx]

Dear Editors,

I am pleased to submit an original qualitative research article entitled "How general practitioners understand and handle medically unexplained symptoms: a focus group study" for consideration for publication in *BMC* *Family Practice*.

Together with my co-author, Karin Isaksson Rø (MD), I explore general practitioners’ (GPs’) approaches to understanding and handling medically unexplained symptoms (MUS). Based on focus group interviews with Norwegian GPs, and using the concept of framing as an analytical lens, we specifically study the role of medical models in framing MUS. We find that the GPs alternated between a biomedical frame, centred on disease, and a biopsychosocial frame, centred on the sick person. Each frame shaped the GPs’ understanding and handling of MUS. The biomedical frame emphasised the lack of objective evidence, problematized subjective patient testimony, and manifested feelings of uncertainty, doubt and powerlessness. This in turn complicated patient handling. In contrast, the biopsychosocial frame emphasised clinical experience, turned patient testimony into a valuable source of information, and manifested feelings of confidence and competence. This in turn made them feel empowered. There was a tendency for GPs with the least experience to rely more on the biomedical frame, whereas their more seasoned seniors tended to rely most on the biopsychosocial frame.

I believe that this manuscript is appropriate for publication in *BMC* *Family Practice* because it addresses important issues within the field of family practice and general medicine. Patients with MUS are a common and challenging encounter, and your journal has published important research articles about MUS – which we engage with in our manuscript. Importantly, in our discussion, we show that the way medical models impinge on GPs understanding and handling of MUS can shed light on findings reported throughout the research literature. It will moreover add to the discussion in the field, I think, to point out how the biomedical model can lead GPs – and junior GPs in particular – to struggle with the considerable distance between the exemplars of their textbooks and clinical reality in primary health care.

This manuscript has not been published and is not under consideration elsewhere, and Karin and I have no conflicts of interest to disclose.

Thank you for your consideration!

Sincerely,

Erik Børve Rasmussen,

MA in sociology and PhD student at the Centre for the Study of Professions,

Oslo and Akershus University College of Applied Sciences, Norway.
